# Supplementary figures and images for: Depletion of Scleraxis-lineage cells during tendon healing transiently impairs multi-scale restoration of tendon structure during early healing
Source: PLoS One. 2022 Oct 14;17(10):e0274227. doi: 10.1371/journal.pone.0274227 (PMC9565440; doi:10.1371/journal.pone.0274227)

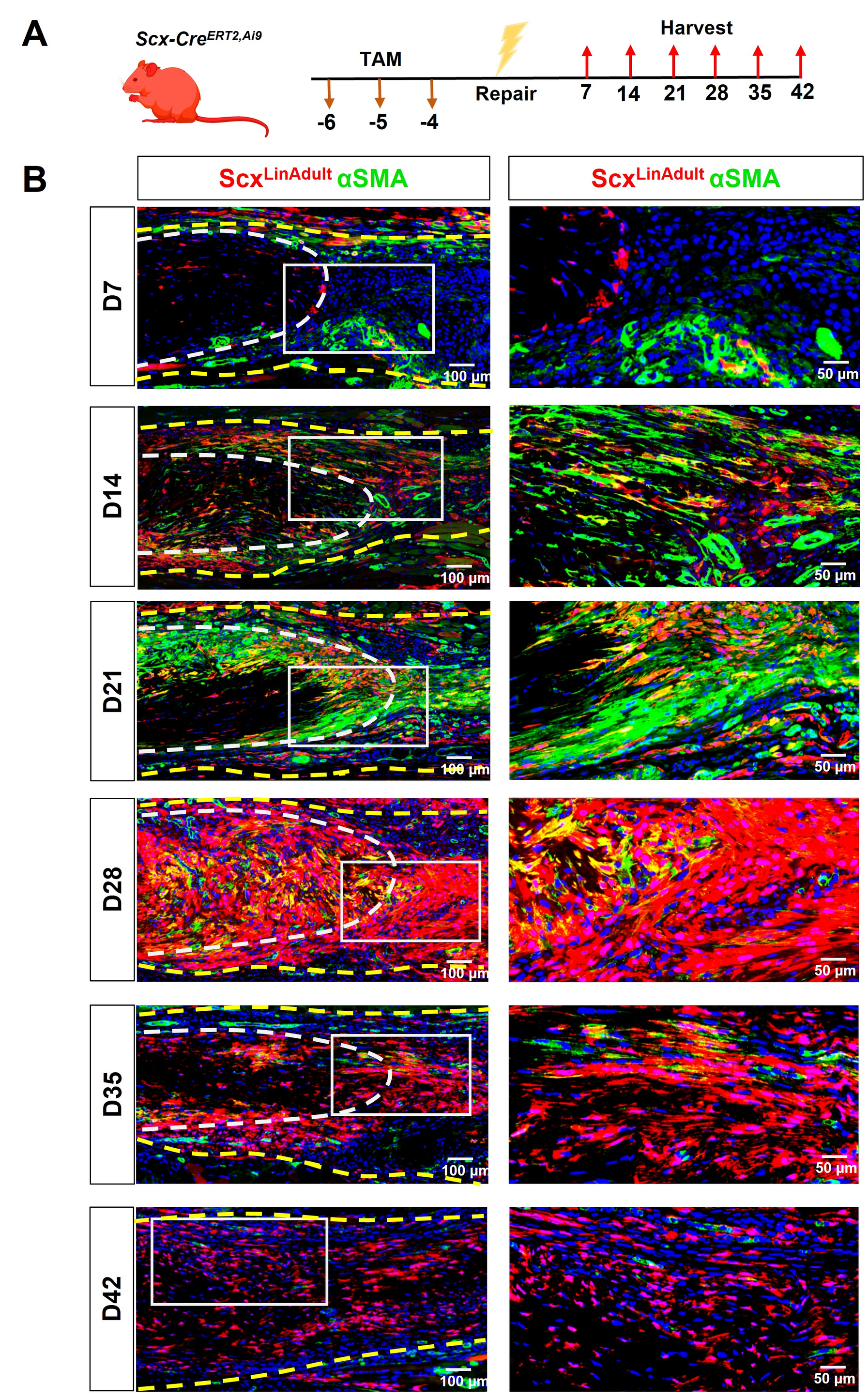

Supplement: S1 Fig — Schematic of the mouse model used and timeline for tamoxifen injections, tendon surgeries, and tissue harvesting. B. Hind paws from Scx-CreERT2,Ai9 mice were probed for Red Fluorescence Protein (RFP) to visualize ScxLinAdult cells, and αSMA-FITC to visualize myofibroblasts. All samples were counterstained with the nuclear dye DAPI. N = 3–5 per timepoint. (TIF) [file pone.0274227.s001.tif]

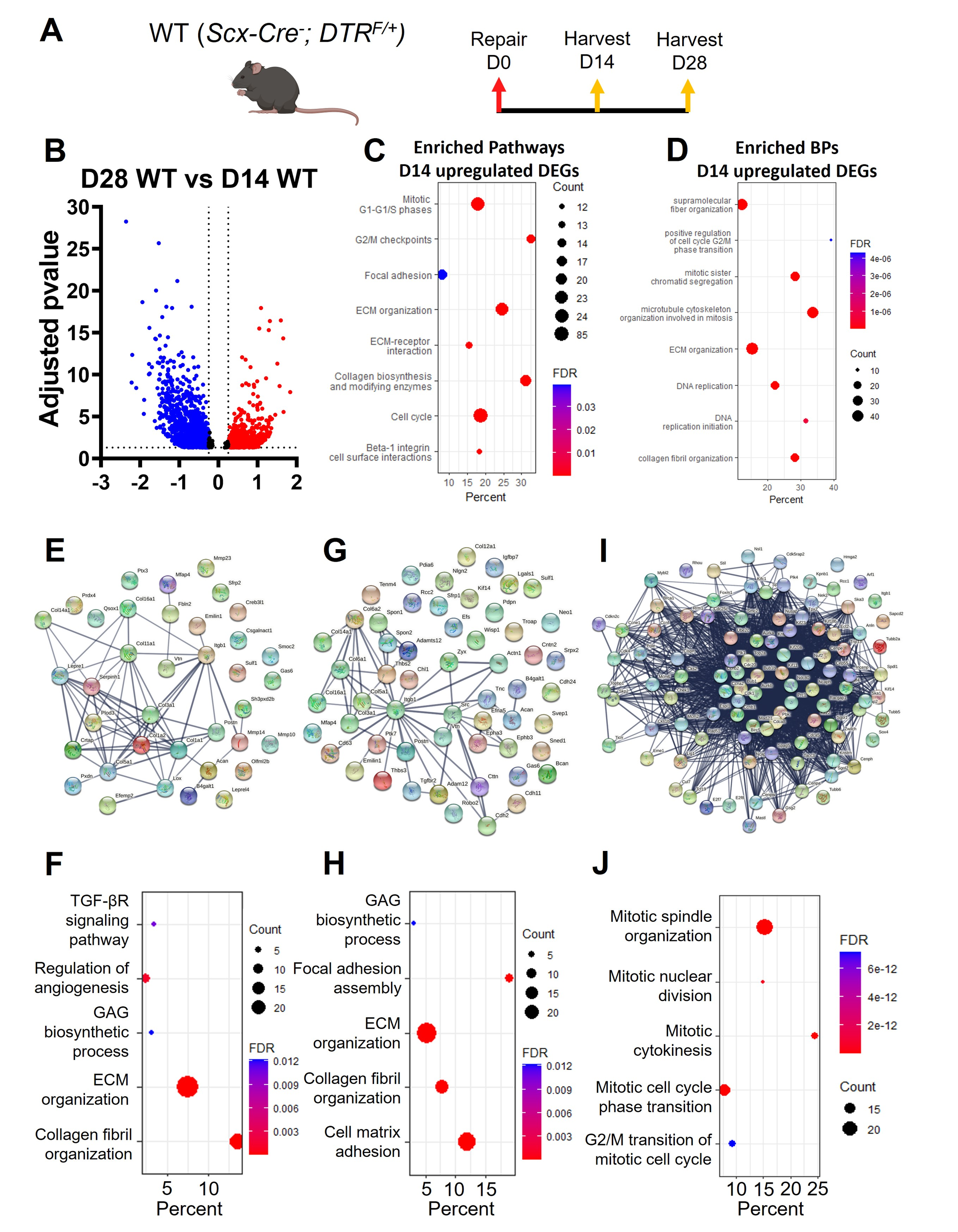

Supplement: S2 Fig — A. Schematic of the mouse model used and timeline for tendon surgeries, DT injections, and tissue harvesting. B. Volcano plot of all the significantly different genes between D28 vs D14 WT tendons. Enriched pathways (C) and biological processess (D) between D28 vs D14 WT tendons. E. Protein-protein communication of all the ECM (E), cell adhesion (G), and cell cycle (I) genes between D28 vs D14 WT tendons. Enriched pathways related ECM (F), cell adhesion (H), and cell cycle (J) between D28 vs D14 WT tendons. (TIF) [file pone.0274227.s002.tif]
